# The early warning paradox

**DOI:** 10.1038/s41746-024-01408-x

**Published:** 2025-02-03

**Authors:** Hugh Logan Ellis, Edward Palmer, James T. Teo, Martin Whyte, Kenneth Rockwood, Zina Ibrahim

**Affiliations:** 1 King’s College London, London, UK; 2 Dalhousie University, Halifax, Canada; 3 Institute of Health Informatics, London, UK; 4 King’s College Hospital NHS Foundation Trust, London, UK; 5 University of Surrey, Guildford, UK

**Keywords:** Outcomes research, Scientific data

## Abstract

Machine learning models in healthcare aim to predict critical outcomes but often overlook existing Early Warning Systems’ impact. Using data from King’s College Hospital, we demonstrate how current evaluation methods can lead to paradoxical results. We discuss challenges in developing ML models from retrospective data and propose a novel approach focused on identifying when patients enter a ‘risk state’ through latent health representations, potentially transforming clinical decision-making.

The advent of machine learning (ML) models in healthcare has ushered in new means to predict critical patient outcomes, such as in-hospital ICU transfer, sepsis or death^1,2^. We highlight an often-overlooked pitfall in the design and evaluation of these models and the role of existing Hospital Early Warning Systems (EWS) in averting the very outcomes they seek to predict.

Traditional EWS are designed to detect patient deterioration in hospitals as a result of underlying physiological dysfunction affecting routinely measured vital signs^3^. While valuable, these systems have limitations: they give no indication of the reversibility of a patient’s state, can generate false alarms, and fail to identify some cases of deterioration^4^. A recent Cochrane Review found low-to-very low certainty evidence on the effectiveness of EWS^5^.

ML architectures aim to address these limitations by leveraging vast amounts of electronic health record (EHR) data. Existing implementations of ML EWS are predominantly supervised ML models that analyse historical data from patients who experienced adverse outcomes. These models identify patterns and indicators in the EHR data that preceded these events. The learned patterns can then be used to detect signs of deterioration before vital signs are sufficiently perturbed to be detected by an EWS.

However, this approach faces two significant impediments. First, there’s a risk of learning bias: if certain patient groups are systematically excluded from specific interventions (e.g. never taken to the ICU), the algorithm may learn and perpetuate this bias^6^. Second, and perhaps more insidiously, a paradox emerges when EWS successfully stabilise at-risk patients.

Consider cases where prompt traditional EWS activation prevents adverse outcomes like sepsis, death, or ICU admission. In these instances, these successfully treated patients become ‘controls’ in a supervised ML model’s training data. We run into the fundamental problem of causal inference: We can only observe one outcome for each patient, leaving us blind to potential alternate scenarios without EWS triggered intervention. Consequently, ML models might inadvertently focus on identifying patients who will deteriorate regardless of intervention, rather than those who could benefit from timely care. This runs counter to our intended goals.

Despite this, within the literature, ML EWS are frequently reported as having superior predictive ability compared with traditional EWS. This alleged superiority is demonstrated using retrospective data, but due to the paradox described above, this approach is flawed. To illustrate this paradox, we conducted an experiment using data from King’s College Hospital, London, where we deliberately compared two single features in isolation: haemoglobin and age. We evaluated their individual abilities to predict 24 h mortality. (Full methods, figures, and results, including ROC plots, are available in the supplementary material; Supplementary Fig. 1).

Low haemoglobin, known as anaemia, can indicate time-critical conditions such as haemorrhage for which effective treatments exist. In this intentionally simplified experiment haemoglobin showed predictive performance for mortality no better than chance. In contrast, age, non-modifiable as far as we know, proved to be a far stronger predictor. Should a clinician at King’s, when called about a patient with anaemia, instead focus on reviewing the oldest patient on the ward? Should a hospital replace their existing EWS with a ML EWS that shows superior predictive ability on retrospective data?

The apparent poor performance of haemoglobin as a predictor demonstrates a crucial point: features and systems linked to effective interventions which succeed in preventing adverse outcomes can appear to have low predictive accuracy in retrospective data. This is because successful interventions alter the outcomes the model aims to predict, weakening the association between predictors and outcomes.

This phenomenon, which Lenert et al. referred to as “prediction decay”, is also observed in well-implemented ML models^7^. Once deployed, they may appear to lose predictive accuracy because the interventions they trigger effectively prevent adverse events. In a utopian scenario, an EWS would show a negative retrospective association with mortality—it would identify only reversible cases of deterioration early enough to avert every preventable death.

To address this challenge and improve model performance and evaluation, one might consider incorporating intervention data. However, this approach, while intuitive, introduces significant complexities. Even in our simple haemoglobin model, including blood transfusion data would lead to confounding by treatment indication and time-dependent confounding. This issue is amplified for an EWS, where various vital signs can be deranged simultaneously and patients often receive several interventions at once. How do we determine which patients were truly at risk? Of the treatments administered, which were necessary, effective and timely?^8^

Again, we are presented with the fundamental problem of causal inference: it is challenging to determine the true effect of interventions from observational data. This challenge is exemplified by two prospective randomised controlled trials (RCTs) of pop-up alerts for patients with acute kidney injury (AKI)^9,10^. In both studies, the alerts measurably changed clinical behaviour: increasing usage of the AKI intervention bundle in the first study and boosting rates of discontinuation of potentially nephrotoxic medications in the second. However, neither study demonstrated a benefit in patient outcomes. More concerningly, the first study revealed significantly higher mortality rates in the intervention group at non-teaching hospitals (15.6% vs 8.6%, *p* = 0.003). These studies highlight that even when models prompt intervention, they may not improve patient outcomes and can potentially lead to harm. Given these challenges and the potential for unintended consequences, the utility of ML models intended for clinical implementation should be rigorously evaluated through RCTs before widespread adoption. This approach is not only feasible, as demonstrated by these AKI studies, but essential to ensure patient safety and clinical efficacy.

How else might we overcome the fundamental impediments in developing EWS using retrospective data and bridge the gap between statistical prediction and causal inference^11^? The outcomes we currently observe are the sequelae for a subset of patients who entered a ‘risk state’ due to physiological insults such as infections, adverse drug reactions, or haemorrhage. If we identify this ‘risk state’ early, we could facilitate timely intervention to prevent the adverse outcome.

To identify this ‘risk state,’ we propose developing a proxy for measuring health - a latent representation inferred from data in EHRs. For instance, Renc et al. create a latent representation of a patient within a transformer model, and can access this hidden representation by asking the model to generate a Sequential Organ Failure Assessment (SOFA) score at any given time for a patient^12^. By monitoring these latent health states, we can identify periods of stable equilibrium and recognize deviations.

This shift in focus from outcome prediction to ‘risk state’ identification addresses the limitations of outcome-based models. Rather than relying solely on supervised ML, we can use our latent representation model to prompt clinician assessment. Incorporating this approach within an RCT would enhance our understanding of clinical actions and help delineate the subset of patients who can benefit from early intervention.

This method allows us to move beyond mere outcome prediction to understanding the mechanisms behind these outcomes. Crucially, it may help distinguish between reversible and irreversible deterioration, enabling more informed decisions about when aggressive treatment is needed and when it might be futile or harmful.

Successful implementation of this novel approach could transform healthcare by enabling earlier, more precise clinical decisions, thereby improving patient outcomes and resource allocation. By deepening our understanding of how and why outcomes occur, we open new avenues for advancing clinical decision-making and patient care.

## Supplementary information

Supplemental Material
