# Supplementary material for: The early warning paradox
Source: NPJ Digit Med. 2025 Feb 3;8:81. doi: 10.1038/s41746-024-01408-x (PMC11790821; doi:10.1038/s41746-024-01408-x)
Supplement: Supplementary file 1 — Supplemental Material [file 41746_2024_1408_MOESM1_ESM.pdf]

Supplementary Information

This note provides detailed methods and results for the example discussed in the main text, where models predicting 24-hour mortality based on haemoglobin and age were compared.

Data Source and Study Population

Data were obtained from the Electronic Patient Records (EPR) and Electronic Prescribing and Medicines Administration (EPMA) systems of King's College Hospital (KCH), London, UK, a large, urban, tertiary-care hospital with 950 beds and four adult critical care units.

The CogStack[1] ecosystem, an information retrieval, extraction, and natural language processing platform, was used to access structured fields in the EHR.

Inclusion and Exclusion Criteria

The study included all hospital admissions to KCH from 1st January 2017 to 31st December 2020 for patients admitted via the emergency department. Patients under 18 years of age or those admitted via other routes were excluded from the analysis.

Ethical Approval

Local approval for the use of Cogstack EPR searches was sought from the King's Electronic Patient Record Interface (KERRI) committee, and approval was received on 11th April 2023 (approval ID 20230411B).

Data Analysis

There were 49,011 hospital admissions meeting criteria during the study period, of which there were 2,322 in-hospital deaths. Analyses were performed using R statistical software, version 3.6.1[2].

Logistic regression models were created to predict 24-hour mortality based on either haemoglobin levels or age. Haemoglobin was chosen as it reflects the oxygen-carrying capacity and volume of blood in the body, with low levels (anaemia) associated with critical conditions requiring immediate attention, such as haemorrhage. Age was selected as a non-modifiable factor for comparison.

The pROC[3] package in R was used to generate ROC curves and calculate the Area Under the ROC Curve (AUROC) for both predictors.

Results

The AUROC for haemoglobin as a predictor of 24-hour mortality was 0.49, indicating performance no better than chance. In contrast, the AUROC for age was 0.65, suggesting a moderately superior predictive power.

These results illustrate the potential disconnect between a feature's statistical predictive power and its clinical utility in identifying patients at risk of deterioration, as discussed in the main text.

Supplementary Figure 1

ROC curves for Haemoglobin and Age as predictors of 24-hour mortality.

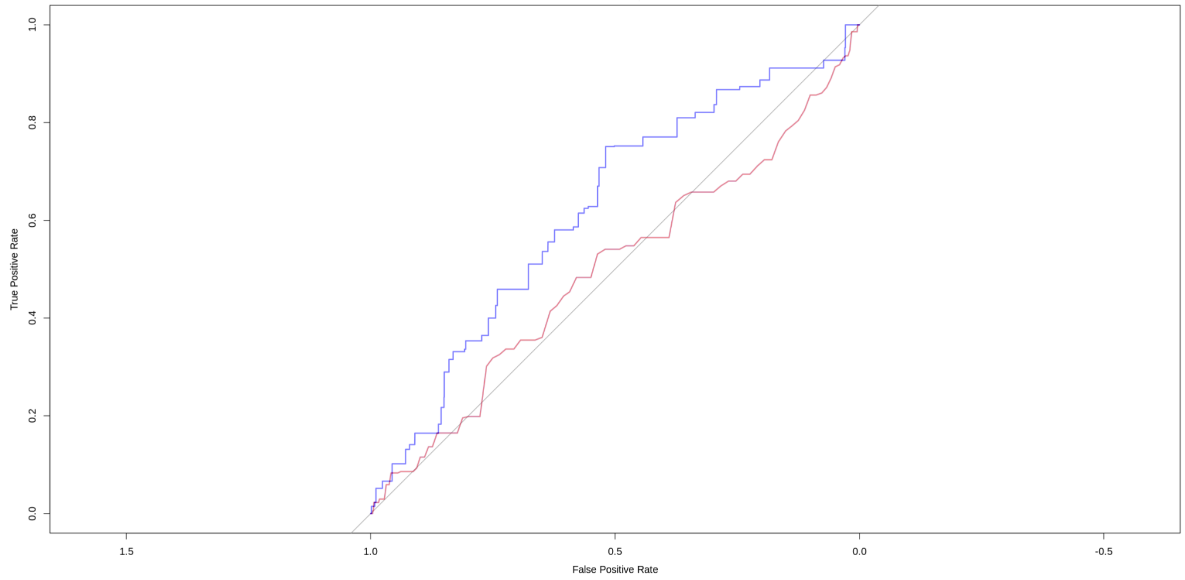

This figure shows the Receiver Operating Characteristic (ROC) curves for Haemoglobin (red) and Age (blue) as predictors of in-hospital mortality within 24 hours. The Area Under the ROC Curve (AUROC) for Haemoglobin is 0.49, indicating performance no better than chance, while the AUROC for Age is 0.65, suggesting moderate predictive power. This illustrates the paradoxical result discussed in the main text, where age, a non-modifiable factor, appears to be a superior predictor compared to haemoglobin, a clinically relevant and potentially modifiable factor.

Supplementary References

1. Jackson, R. *et al.* CogStack - experiences of deploying integrated information retrieval and extraction services in a large National Health Service Foundation Trust hospital. *BMC Med. Inform. Decis. Mak.* **18**, (2018).
2. R Core Team. (2023). R: A language and environment for statistical computing. R Foundation for Statistical Computing, Vienna, Austria. URL https://www.R-project.org/.
3. Robin, X. *et al.* pROC: an open-source package for R and S+ to analyze and compare ROC curves. *BMC Bioinformatics* **12**, 77 (2011).
